# Neither action nor phonological video games make dyslexic children read better

**DOI:** 10.1038/s41598-017-18878-7

**Published:** 2018-01-11

**Authors:** Magdalena Łuniewska, Katarzyna Chyl, Agnieszka Dębska, Agnieszka Kacprzak, Joanna Plewko, Marcin Szczerbiński, Jakub Szewczyk, Anna Grabowska, Katarzyna Jednoróg

**Affiliations:** 1 Laboratory of Psychophysiology, Department of Neurophysiology, Nencki Institute of Experimental Biology, Polish Academy of Sciences, Warsaw, Poland; 2 School of Applied Psychology, University College Cork, Cork, Ireland; 3 Institute of Psychology, Psychology of Language and Bilingualism Lab, Jagiellonian University, Kraków, Poland; 4 Faculty of Psychology, University of Social Sciences and Humanities, Warsaw, Poland

## Abstract

The prevalence and long-term consequences of dyslexia make it crucial to look for effective and efficient ways of its therapy. Action video games (AVG) were implied as a possible remedy for difficulties in reading in Italian and English-speaking children. However, the studies examining the effectiveness of AVG application in dyslexia suffered from significant methodological weaknesses such as small sample sizes and lack of a control group with no intervention. In our study, we tested how two forms of training: based on AVG and on phonological non-action video games (PNAVG), affect reading in a group of fifty-four Polish children with dyslexia. Both speed and accuracy of reading increased in AVG as much as in PNAVG group. Moreover, both groups improved in phonological awareness, selective attention and rapid naming. Critically, the reading progress in the two groups did not differ from a dyslexic control group which did not participate in any training. Thus, the observed improvement in reading in AVG and PNAVG can be attributed either to the normal reading development related to schooling or to test practice effect. Overall, we failed to replicate previous studies: Neither AVG nor PNAVG remedy difficulties in reading in school children.

## Introduction

Dyslexia is defined as a specific impairment of reading acquisition which occurs despite typical intelligence and sufficient educational resources^1^. In addition to obvious reading difficulties and long-term influence on academic achievement^2^, dyslexia may lead to social problems, lower self-esteem and higher depression scores^3^, as well as to higher risk for psychiatric problems^4^. Reading may remain impaired also in adulthood^5^ and not improve spontaneously. Because of relatively low chances for spontaneous improvement, children with dyslexia should be properly supported in literacy acquisition. However successful dyslexia remediation is still very far from being fully achieved^6,7^.

The most widely accepted cognitive cause of dyslexia is a deficit of phonological processing^1,8^, i.e. a difficulty in efficient processing of speech sounds. The deficit of phonological processing manifests itself across various tasks such as phonological awareness (phoneme identification, analysis or blending), phonological short-term memory (pseudoword repetition), learning of new words, or word retrieval (naming)^9,10^. Since efficient phonological processing is crucial for reading acquisition^11^, several studies aimed to check whether a phonology-based training may result in improved phonological processing and whether this enhancement may be transferred to reading progress. As expected, phonological training led to increase of phonological awareness and reading outcomes both in children with dyslexia and at risk for reading impairment^12–16^.

Alternatively, it has been proposed that dyslexia stems from an attentional dysfunction^17,18^. According to this hypothesis, reading needs efficient serial processing of stimuli which may be achieved by rapid orientation of visual attention^19^. Thus, efficient visual attention skills are necessary for fluent reading. The visual attention hypothesis was supported by two interventional studies^19,20^ in which dyslexic children who played video games with high attentional requirements (action video games, AVG) significantly improved their reading skills, in contrast to peers who played non-action video games (NAVG) and who made no progress in reading. The authors concluded that trainings based on AVG enhance visual attention and therefore lead to an improvement of both reading speed and accuracy. However, both studies involved only small samples of participants (Italian: N = 20; English: N = 28) and thus were seriously underpowered^21^. Therefore, the idea that AVG may provide a successful tool for dyslexia remediation needs further verification on a bigger sample.

The study presented here aimed to examine the effectiveness of attentional and phonological trainings in Polish children with dyslexia. Previous research on symptoms of dyslexia in Polish replicated the phenomena known from other languages^22^: Polish dyslexic children struggle with both reading accuracy and speed^22–24^, and a fraction of them presents deficits in phonological awareness^25^, as well as in visual attention^26^. We applied two training methods: one based on action video games (AVG) and one based on phonological non-action video games (PNAVG). The first training method was the same as used by Franceschini *et al*.^19,20^. This enabled us to verify results showing that AVG improve reading skills children with dyslexia. The second training method, PNAVG, was different than in previous studies. While the NAVG applied in the previous studies was deliberately chosen as a control condition with no impact on reading, we decided to use a NAVG which potentially could affect reading positively. Since phonological awareness training was shown to enhance reading in children^27^, we designed and employed a NAVG based on phonological awareness tasks. We carefully selected purely phonological tasks as minigames in PNAVG, in order to train phonological awareness. Inclusion of PNAVG group enabled us to verify results of several prior studies which showed that phonology-based trainings transfer to reading progress in children with dyslexia and at risk for reading impairment^12–16^.

In addition to the two groups who played AVG or PNAVG, we included a control group who did not participate in any video-game-based training. Similarly to both AVG and PNAVG groups, the control group consisted of children with dyslexia. The three groups completed the same series of web-based tasks of reading-related skills. We expected that the effectiveness of both interventions would be reflected in the rates of improvement in the reading-related tasks. Namely, we anticipated that the progress made by the two training groups would be bigger than the one of the non-training dyslexic control group.

## Results

### The impact of training on reading performance: AVG vs. PNAVG comparison

We used two measures of reading skills: the number of correctly read items (words or pseudowords) per minute, and an index of reading inefficiency based on Franceschini *et al*.^19^. The index of reading inefficiency was measured as the ratio between speed (time in seconds necessary to read an item) and accuracy (percentage of correct responses).

We performed two analyses of variance (ANOVAs) with either the number of items correctly read per minute or the reading inefficiency as dependent variables. We used a mixed design including two within-subject factors of Task (word vs. pseudoword reading) and Time (before vs. after training), and a between-subject factor of Group (AVG vs. PNAVG).

**Figure 1:**
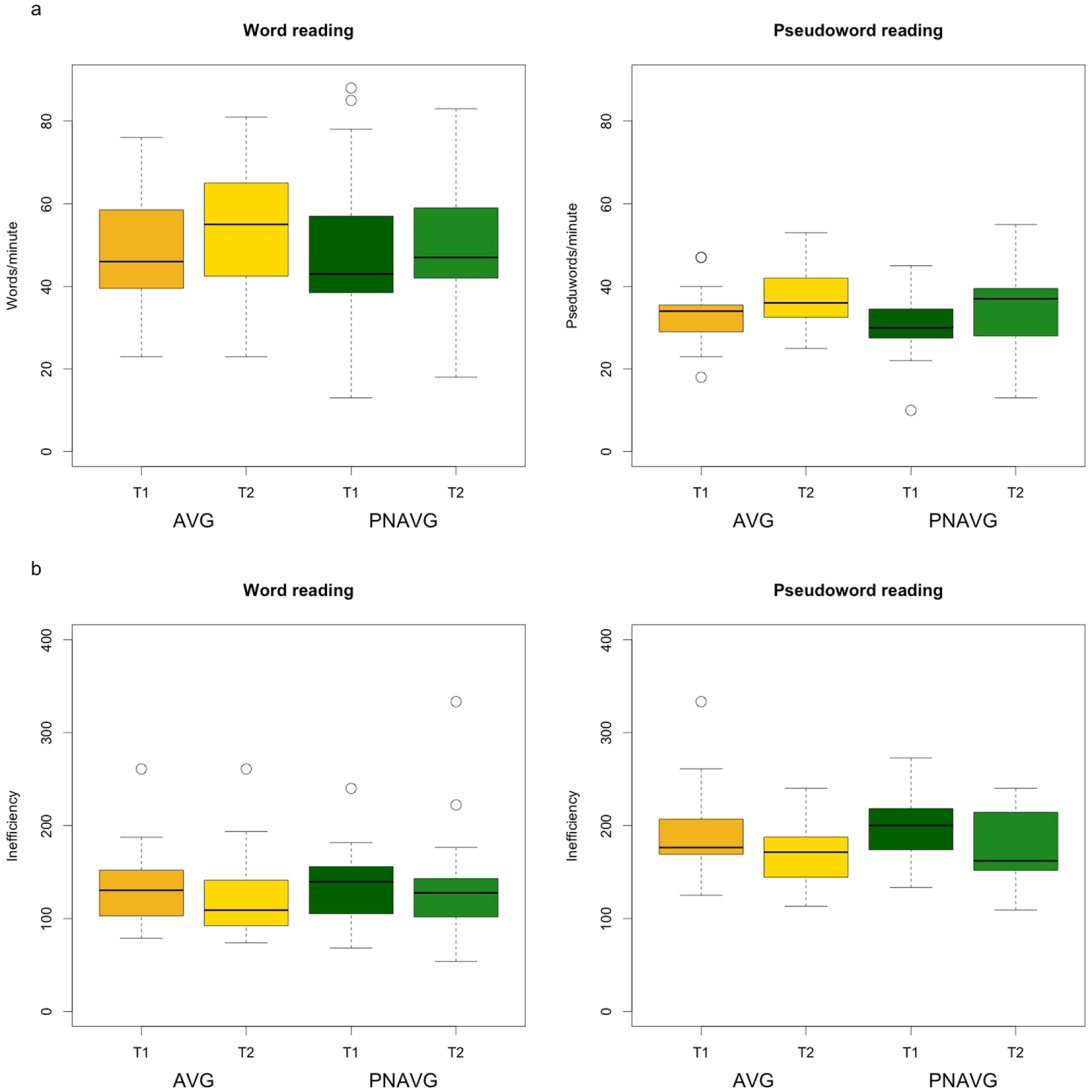
Reading scores (**a**: correctly read items per minute, **b**: reading inefficiency index) in word and pseudoword reading across AVG and PNAVG groups before (T1) and after (T2) the training.

For the number of correctly read items, we found significant main effect of Task (F(1,52) = 110.34, p < 0.001, η^2^ = 0.68) and main effect of Time (F(1,52) = 41.82, p < 0.001, η^2^ = 0.45; see Fig. 1a). Children read more words than pseudowords, and after the training they were able to read more items per minute than before it. Neither Group effect (F(1,52) = 0.29, p = 0.60) nor interactions of Group * Time (F(1,52) = 0.29, p = 0.59), Group * Task (F(1,52) = 0.02, p = 0.90) and Time * Task (F(1,52) = 0.09, p = 0.77) were significant.

With respect to the index of reading inefficiency index, we found main effects of Task (F(1,52) = 210.26, p < 0.001, η^2^ = 0.80) and Time (F(1,52) = 34.08, p < 0.001, η^2^ = 0.40), as well as a Task * Time interaction (F(1,52) = 12.95, p = 0.001, η^2^ = 0.20; see Fig. 1b). The reading inefficiency scores were lower after the training than before it, i.e. reading was more efficient after the training. Pseudoword reading was less efficient than word reading, and improved more with the time. Neither Group effect (F(1,52) = 1.10, p = 0.30) nor interactions of Group * Time (F(1,52) = 0.13, p = 0.72) and Group * Task (F(1,52) = 0.74, p = 0.39) were significant.

Concluding, type of training did not have any influence on either of the reading scores.

### The impact of training on reading performance: Individual results

**Figure 2:**
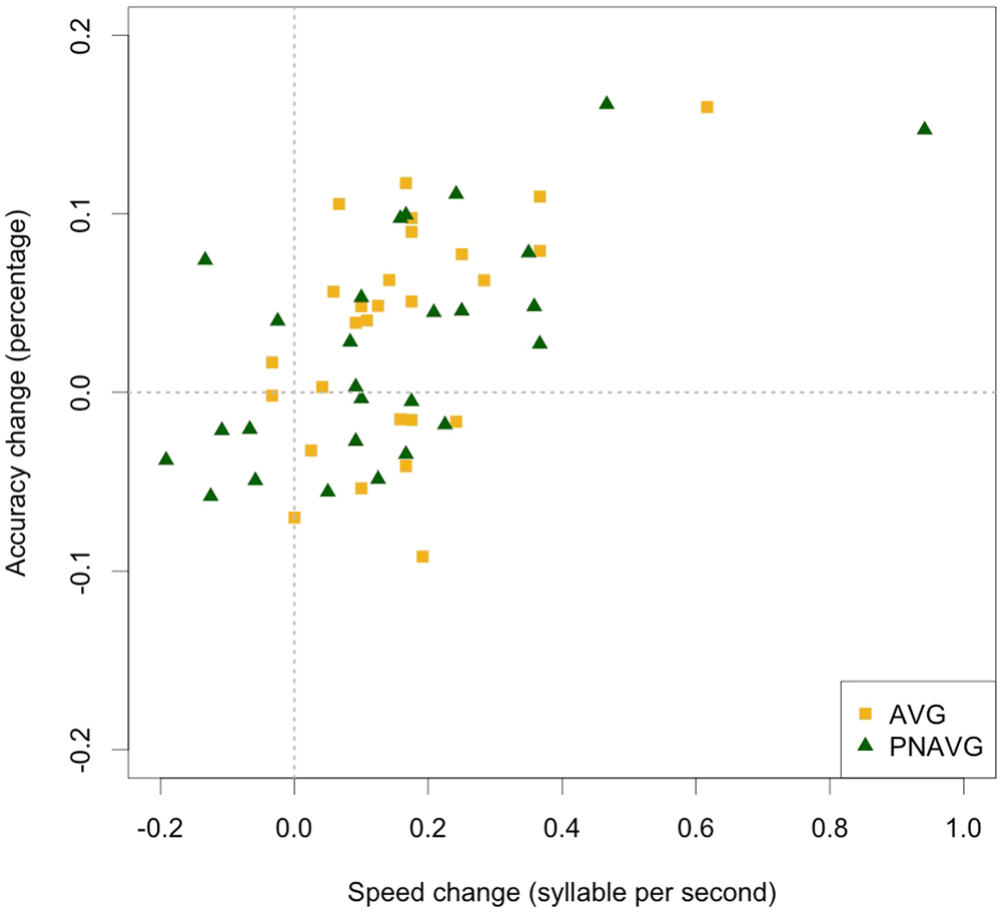
The mean of word and pseudoword reading speed (in syllable per second) and accuracy (percentage of correctly read items) change between T1 and T2 reported for each child of the AVG (yellow squares) and PNAVG (green triangles). The upper right corner contains the participants who in T2 scored higher in both reading speed and accuracy than in T1.

Following Franceschini *et al*.^20^, we also tested the effect of training on reading speed and accuracy on an individual level. Changes in reading speed (mean of difference between T2 and T1 for words and pseudowords, reported in syllables per second) and accuracy (mean of difference between T2 and T1 for words and pseudowords, reported in percentage of correct to total of read items) are reported in Fig. 2. Following Franceschini *et al*.^20^, we calculated how many children in the AVG and PNAVG groups showed improvement in reading during the training. Similarly to Francechini *et al*.^20^, we assumed that children who read more syllables per second in T2 than in T1 increased their reading speed, and children who made less errors, i.e. read relatively more words correctly, made progress in reading accuracy. Conversely, children who either presented the same level of reading skills in T1 and T2, or showed lower performance in T2 than in T1, were labelled as those who made no progress in reading.

An increase of reading speed was observed in 82% of the whole sample: 89% of AVG group and 74% of PNAVG. Improvement of reading accuracy was present in 61% children: 67% of AVG and 56% of PNAVG. Joint enhancement of reading speed and accuracy occurred in 56% of children: 63% of AVG and 48% of PNAVG. The two trainings did not differ in the proportion of children who experienced progress in reading (speed: χ^2^(1, N = 54) = 1.96, p = 0.15; accuracy: χ^2^(1, N = 54) = 0.70, p = 0.29; both accuracy and speed: χ^2^(1, N = 54) = 1.20, p = 0.21).

### The impact of training on non-reading skills

The aim of this analysis was to check whether the training led to enhancement of non-reading skills, such as phonological awareness, phonological working memory and sublexical knowledge, selective visual attention and rapid naming.

#### Phonological awareness

**Figure 3:**
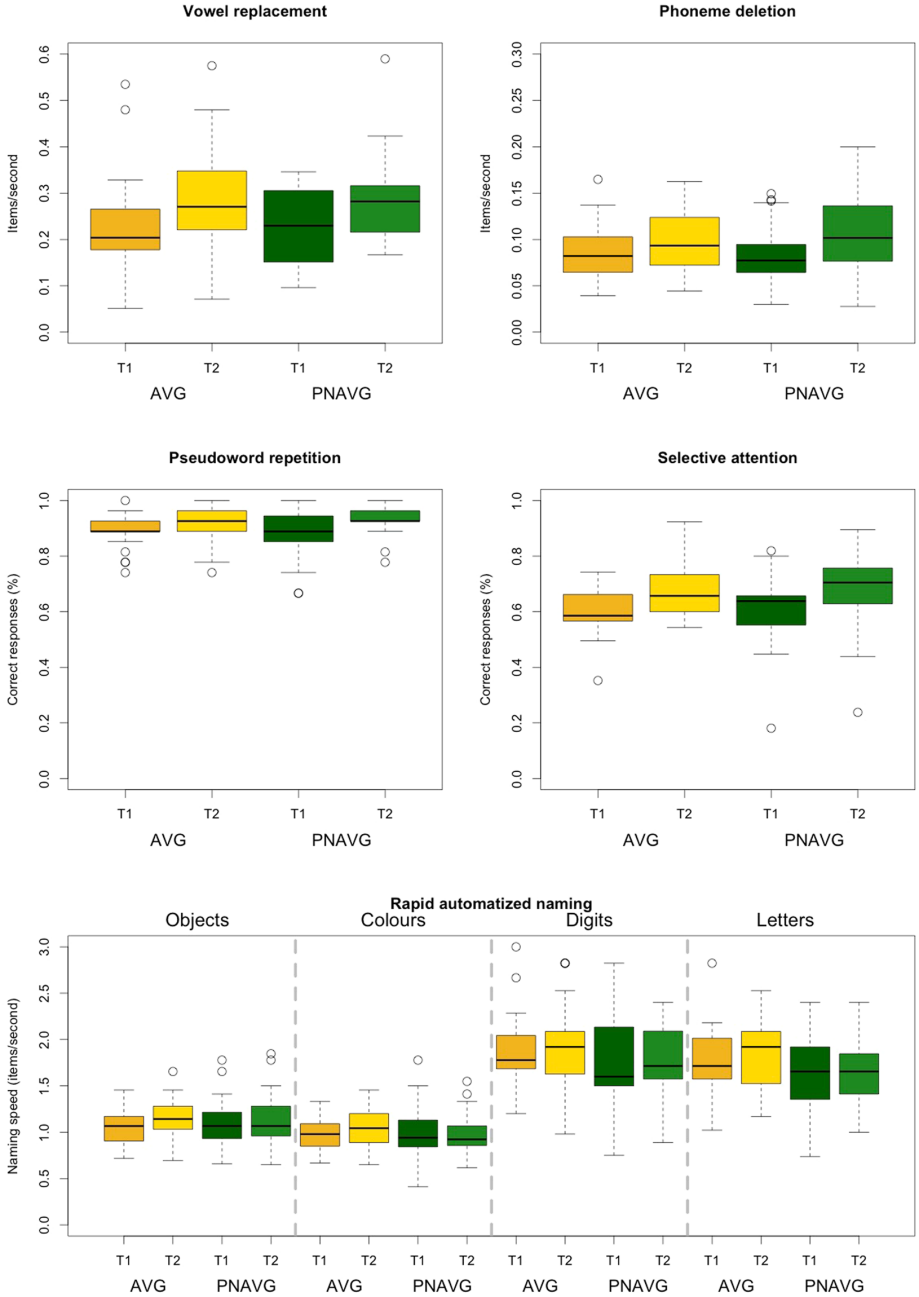
Performance in phonological, attentional and rapid naming tasks before and after the training. In all measures, the effect of Time was significant, but there are no effects of Group nor Group * Time interaction.

In order to control for the trade-off between speed and accuracy in phonological awareness task, for both tasks (phoneme deletion and vowel replacement) we calculated the number of correctly solved items per second. Changes in phonological awareness were analysed using two 2 × 2 mixed-design ANOVAs with a within-subject factor of Time (before vs. after training) and a between-subject factor of Group (AVG vs. PNAVG), with the solved items per second as the dependent variable, separately for phoneme deletion and vowel replacement. For phoneme deletion, we found a significant effect of Time (F(1,50) = 17.96, p < 0.001, η^2^ = 0.26, see Fig. 3). Neither the Group effect (F(1,50) = 0.01, p = 0.91) nor the Group * Time interaction (F(1,50) = 1.78, p = 0.19) were significant. Similarly, for vowel replacement we found a significant effect of Time (F(1,49) = 30.77, p < 0.001, η^2^ = 0.39), but no effect of Group (F(1,49) < 0.01, p = 0.97) nor Group * Time interaction (F(1,49) < 0.01, p = 0.99). In both phonological awareness tasks participants scored higher after the training than before it, regardless of the type of training.

#### Phonological working memory and sublexical knowledge

Changes in phonological working memory and in sublexical knowledge were measured with accuracy in a pseudoword repetition task. They were analysed using a 2 × 2 mixed-design ANOVA, with a within-subject factor of Time (before vs. after training) and a between-subject factor of Group (AVG vs. PNAVG), with raw score in pseudoword repetition task as the dependent variable. The main effect of Time (F(1,52) = 15.53, p < 0.001, η^2^ = 0.23) was significant (see Fig. 3), indicating that after the training participants achieved higher scores than before. Neither the Group effect (F(1,52) = 0.01, p = 0.94) nor the Group * Time interaction (F(1,52) = 0.34, p = 0.56) were significant.

#### Selective attention

Changes in visual selective attention were assessed with raw score (percentage of obtained points) in a selective attention subtest of IDS Intelligence Scale^28^. Outcomes were analysed using 2 × 2 mixed-design ANOVA, with a within-subject factor of Time (before vs. after training) and a between-subject factor of Group (AVG vs. PNAVG), with a percentage score in selective attention task as the dependent variable.

The main effect of Time (F(1,44) = 96.56, p < 0.001, η^2^ = 0.69) was significant (see Fig. 3), indicating that after the training children achieved higher scores than before it. Neither the Group effect (F(1,44) = 0.04, p = 0.94) nor the Group * Time interaction (F(1,44) = 0.03, p = 0.86) were significant.

#### Rapid automatized naming

Changes in rapid automatized naming measured with four rapid naming subtests were analysed using a 4 × 2 × 2 mixed-design ANOVA with a within-subject factors of Stimuli type (objects vs. colours vs. digits vs. letters) and Time (before vs. after the training), and a between-subject factor of Group (AVG vs. PNAVG), and items named per second as the dependent variable. The main effects of Time (F(1,52) = 6.73, p = 0.01, η^2^ = 0.12) and Stimuli type (F(3,50) = 130.62, p < 0.001, η^2^ = 0.89) were significant (see Fig. 3). After the training children were able to name the items faster than before it, and naming of digits and letters was faster than naming of objects and colours. Neither the Group effect (F(1,52) = 1.06, p = 0.31) nor any type of interaction, i.e. Group * Time interaction (F(1,52) = 2.60, p = 0.12), Group * Stimuli (F(3,50) = 1.21, p = 0.31), Time * Stimuli (F(3,50) = 0.77, p = 0.52), were significant.

### Reading improvements as compared to the dyslexic control (no-training) group

**Figure 4:**
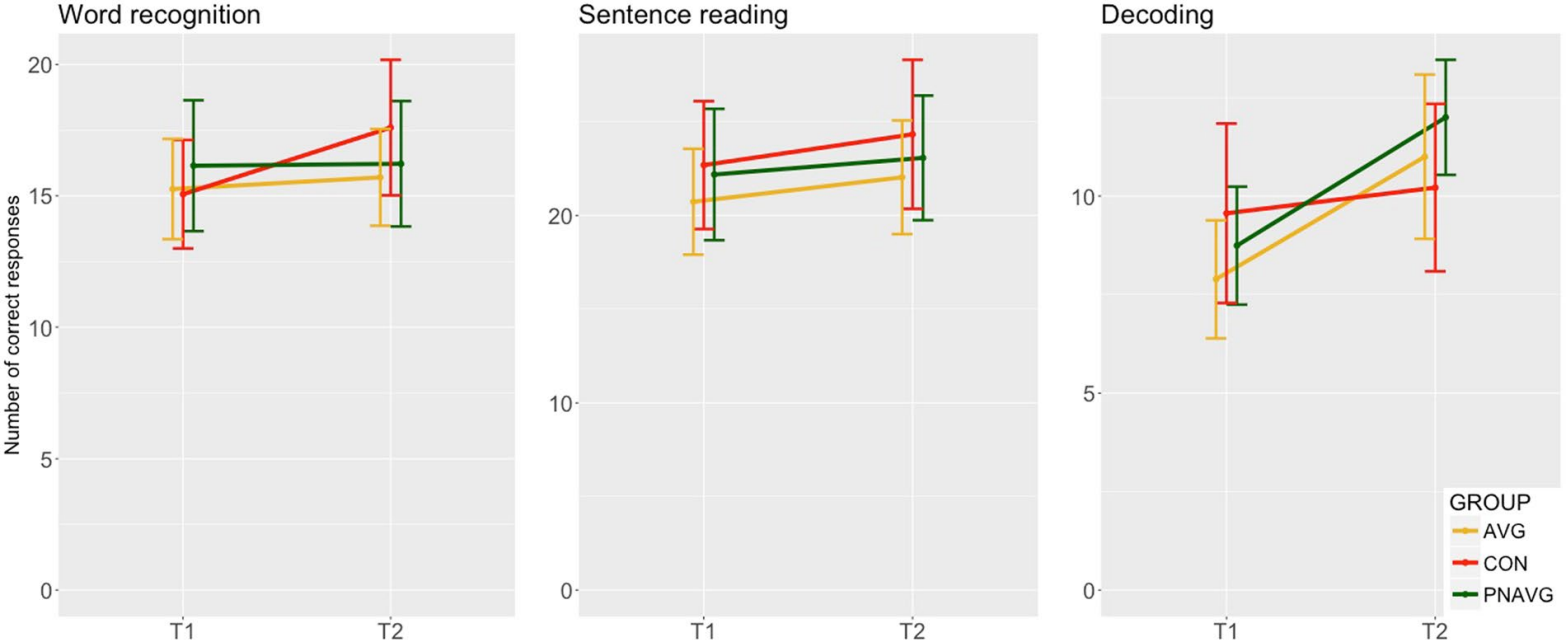
Progress in online reading tasks between T1 (before the training) and T2 (after the training) in AVG (yellow line), PNAVG (green line) and control group (red line). Error bars correspond to 95% confidence intervals. In sentence reading and decoding there was an effect of Time but there was neither effect of Group nor Group * Time interaction.

The aim of this analysis was to verify whether the reading improvements observed both in the AVG and PNAVG groups constitute specific effects of training, beyond reading progress made by the dyslexic control group which did not participate in the training program. Performance on three reading-related skills measured with a web-based test battery: (1) word recognition, (2) speed of sentence reading, and (3) speed of decoding was analysed separately for each task with a three 2 × 3 ANOVAs with a within-subject factor of Time (before vs. after the training) and a between-subject factor of Group (AVG vs. PNAVG vs. CON), and the number of correct responses given in a limited time in each task as the dependent variable. For word recognition, we found no significant effects (Time: F(1,66) = 3.12, p = 0.08; Group: F(2,66) = 0.17, p = 0.84; Group * Time: F(2,66) = 1.41, p = 0.25, see Fig. 4). For speed of sentence reading, we found a significant effect of Time (F(1,65) = 4.03, p = 0.049, η^2^ = 0.06): the scores increased with time. There was neither the effect of Group (F(2,65) = 0.33, p 0.72) nor the Group * Time interaction (F(2,65) = 0.08, p = 0.92). For decoding speed, we found an effect of Time (F(1,65) = 9.67, p = 0.003, η^2^ = 0.13): children scored higher at the second measurement. There was neither the effect of Group (F(2,65) = 0.43, p = 0.65) nor the Group * Time interaction (F(2,65) = 2.39, p = 0.10). Concluding, in two out of three tasks the scores increased with the time, but there was no difference between the training and the control groups.

The same pattern (the significant effect of Time, but no significant effect of Group, nor Group * Time interaction) was also found when additional data from pre-test (a month before the training) and post-test (a month after the training) were included (see Supplementary materials 4).

## Discussion

Since dyslexia may have serious impact on educational outcomes and wellbeing of affected children^2–5^ there is a need for effective remediation methods. Since children are typically eager to play computer games^29^, a training programme based on video games could be a preferred by children solution for reading difficulties.

Previous studies^19,20^ brought some evidence for efficiency of AVG training in Italian and English speaking children with reading difficulties. Both studies found that children playing AVG improved reading, whereas the reading skills of children playing NAVG remained unchanged. However, the conclusions driven from these studies were based on data from small groups of participants (Italian: N = 20; English: N = 28), raising the possibility that they were due to a sampling error. Here we aimed to verify the hypothesis concerning enhancement of reading after AVG training in a bigger group of Polish-speaking children (N = 54). With such number of participants, we obtained over 99% statistical power to find an effect of training of a previously reported size^19,20^. Also, the sample size gave us 99% power to find a medium-size effect and 52% to find a small effect, according to power analyses done with a use of GPower^30,31^, whereas the corresponding powers in a study with total of 20 participants are 88% for a medium-size effect and 14% for a small effect. As another branch of research showed that phonological awareness trainings improved both phonological awareness itself and reading in children with dyslexia and at risk for reading impairment^12–15^, we designed phonological awareness non-action video games, and compared its effectiveness to the one of AVG.

We found that AVG and PNAVG trainings resulted in the increase of both reading accuracy and speed (see Figs 1 and 2). Progress in reading speed was observed in 82% of children, while progress in reading accuracy was observed in 61% of participants. However, a dyslexic control group, who did not participate in any special training, presented the same enhancement or stability of reading-related skills as the two training groups (see Fig. 4 and Supplementary materials 4). The absence of a between-group difference in the magnitude of reading progress suggests that the increase observed in the training groups stemmed from the repeated measurement, a task learning effect or schooling rather than from real effectiveness of applied trainings. This finding throws into question previously reported effectiveness of AVG trainings and some aspects of the phonological awareness trainings, especially in the light of relatively high statistical power of the current study.

In addition to the improvement in reading across both AVG and PNAVG groups, we observed an enhancement in performance in many other tasks, such as tests of phonological awareness, phonological working memory and sublexical knowledge, selective attention and rapid naming (see Fig. 3). We expected to find a training-specific boost of skills, i.e. an increase of phonological awareness in PNAVG group and an increase of attention in AVG players. However, the observed effects were non-specific: the enhancement was present in both groups to the same extent. The increase of attention and phonological awareness in both groups suggests that this progress reflects merely the test practice effect or regular development of cognitive skills. The observed increase of rapid naming, a skill previously shown to be difficult to train^32^, suggests that we obtained rather a test practice effect than an objective enhancement of skills. The average time between the first and the second testing was 46 days which is both short enough for children to remember the tasks on one hand, and long enough to develop skills due to schooling on the other hand. Although the reading and phonological tasks applied in testing before and after the training used different items (see Supplementary materials 1), the situation of the testing session was the same in both time points, i.e. the testing environment and the procedure did not differ between the measurements. Possibly children partially remembered the tasks and thus used better strategies of solving them during the second testing.

AVG and PNAVG trainings were ineffective in terms of both: their impact on domain-specific skills and their impact on reading skills. There are several possible reasons for the observed ineffectiveness of the trainings, which concern both the training procedure itself as well as the characteristics of its participants. Although the AVG training used exactly the same games as in the previous studies^19,20^, the form of playing differed. In the previously reported studies, participants played the games on a Wii console with a remote controller. Here we used standard notebooks and computer mouses instead, as this equipment is far more common in households. Potentially the difference in the way of controlling the game could affect the results: previously employed AVG training involved body movement during the play, while participants of the current study were seated without any particular movement. On the other hand, if it was the motor component of the games that led to increase of visual attention and reading, its effect would be also visible in NAVG group who used the same equipment for playing. The two AVG trainings – the original one as described by Franceschini *et al*.^19,20^ and the one applied in the current study – differed also in intensity. The previously reported trainings lasted two weeks and consisted of nine 80-minute-long sessions (720 minutes in total), whereas both trainings used in the current study were less condensed and included 16 sessions of 50 minutes of playing (800 minutes in total) in four weeks. However, as participants of the current study spent more time on training, it should rather result in more pronounced progress, as compared to the shorter training in the study by Franceschini *et al*.^19,20^. What is more, the task applied for the assessment of selective attention was quite far from the trained skills, as it did not involve reacting to rapidly appearing stimuli, however previous research brought evidence also for far transfer of AVG effects to visual selective attention^33,34^. Possibly, the applied training improved other aspects of attention such as spatial or temporal attention as reported by Franceschini *at al*.^19^, but these domains of visual attention were out of scope of the current paper.

The observed ineffectiveness of the phonological awareness (PNAVG) training designed specifically for the aims of current study is perhaps more surprising than the ineffectiveness of NAVG training used in previous research, since the positive impact of phonological awareness training on reading has been demonstrated consistently in previous studies^27,35^. We see several potential reasons of the ineffectiveness of the applied PNAVG training, mostly related to its specific design. Firstly, as we aimed at training phonological awareness and not reading itself, most of phonological awareness tasks used in our PNAVG training were ‘pure’, i.e. only sound-based, without any print component (see Supplementary materials 3). Only one task of six required some phoneme-to-grapheme mapping (typing of single letters or syllables in response to auditory input). Since phonological awareness training which does involve letters tends to have greater impact on reading skills^27,35^, it is possible that including a letter component in all of our phonological awareness training tasks would render our PNAVG training more effective. Secondly, in order to provide the participants with various stimuli and grab their attention, we trained several different phonological awareness skills (alliterations, phoneme and syllable blending, phoneme and syllable deletion, rhyming), while it may be the case that focussing on fewer phonological skills, those clearly related to the demands of reading and spelling (such as analysis and blending), as well as involving phonemes only (as opposed to syllables and rhymes) may be more beneficial^35,36^. Lastly, due to technical aspects of video games, we used input phonological tasks, i.e. requiring nonverbal response (pointing and clicking) without overt generation of speech. Possibly a training involving phonological output tasks, requiring the production of oral response, would be more stimulating and thus more effective. Also here the applied measurement of phonological awareness differed from the exact tasks used during the training. Perhaps measurement of the performance in the particular tasks applied in the training would result in observing a training-specific increase of skills.

Finally, despite their dyslexia, the participants of the current study were relatively experienced readers with at least three years of formal reading instruction. It is possible that the applied trainings would be more effective if used in a group of less experienced readers at the very beginning of formal literacy instruction. This applies to the phonological awareness intervention in particular, as especially phonological awareness interventions were shown to be of use in preschool children^14,27^. However, this is not the case of the AVG training, as the previously tested children were roughly at the same age (mean age around 10 years)^19,20^ as the participants of the current study (mean age 11.0 years).

Current study questions the effectiveness of AVG and video games based on pure phonological awareness training as intervention programme for school children with developmental dyslexia. As the trainings were ineffective in terms of both their impact on reading as well as their impact on the particular trained skills, we speculate that not only obtaining a far transfer from the trained skill to reading is difficult, but also obtaining a close transfer to another skill of the same domain may be impossible with a training based on video games. At the same time, further studies on even bigger samples than ours are still needed to reveal potential factors influencing the effectiveness of training programmes in dyslexia.

## Methods

### Participants

#### Training groups

Fifty-four dyslexic children (36 boys, 18 girls), mean age 11.0 years (range 9.0–13.2) participated in the experiment. All the participants were recruited through schools (parental gatherings) or project website. Before the training, all the children took part in a broader study on dyslexia, which involved dyslexia diagnosis, IQ assessment, assessment of attentional and auditory skills via computerized tasks and both functional and structural magnetic resonance (MRI). The study was approved by Research Ethics Committee at the SWPS University of Social Sciences and Humanities in Warsaw and carried out in accordance the provisions of the World Medical Association Declaration of Helsinki. Parents of children who participated signed an informed consent form.

Inclusion criteria for participation in the training were: (i) diagnosis of dyslexia confirmed with a standardized battery of tools performed by an experimenter involved in the project, (ii) no history of psychiatric or neurological disease, (iii) IQ ≥ 85, (iv) normal or corrected to normal vision, (v) no ADHD diagnosis, (vi) ability to arrive to the place of training for 18 times in six weeks (which, in practice, implied living in or around the city of Warsaw, where the training was conducted).

Children were randomly allocated to either AVG (n = 27) or PNAVG training (n = 27), see Table 1 for details.

**Table 1:** AVG and PNAVG group characteristics. Mean (SD).

	AVG (n = 27)	PNAVG (n = 27)	
Gender	Female: 9, Male: 18	Female: 9, Male: 18	χ^2^(1) = 0.00, p = 1.00
School grade	3^rd^ grade: 2, 4^th^ grade: 15, 5^th^ grade: 5, 6^th^ grade: 5	3^rd^ grade: 3, 4^th^ grade: 9, 5^th^ grade: 10, 6^th^ grade: 5	χ^2^(3) = 3.37, p = 0.34
Age (years)	11.04 (1.00)	10.96 (1.00)	t(52) = 0.28, p = 0.79
Word list reading (items read correctly in a minute)	49.15 (13.85)	48.59 (18.05)	t(52) = 0.13, p = 0.90
Pseudoword list reading (items read correctly in a minute)	32.67 (6.63)	30.67 (7.38)	t(52) = 1.05, p = 0.30
Phoneme deletion (correct responses, max = 16)	10.93 (2.77)	10.96 (2.90)	t(52) = −0.05, p = 0.96
Phoneme deletion (time to solve 16 items, in seconds)	133.63 (30.31)	142.23 (46.85)	t(52) = −0.80, p = 0.43
Vowel replacement (correct responses, max = 24)	18.59 (5.83)	19.80 (3.24)	t(52) = −0.91, p = 0.37
Vowel replacement (time to solve 16 items, in seconds)	91.48 (26.07)	98.32 (33.52)	t(52) = −0.83, p = 0.41
Pseudoword repetition (correct responses, max = 27)	24.15 (1.66)	23.96 (2.50)	t(52) = 0.32, p = 0.75
Rapid automatized naming: object (time in seconds)	46.48 (9.18)	46.48 (11.42)	t(52) = 0.00, p = 1.00
Rapid automatized naming: colours (time in seconds)	50.37 (8.78)	52.48 (17.10)	t(52) = −0.57, p = 0.57
Rapid automatized naming: digits (time in seconds)	26.67 (5.23)	29.19 (9.21)	t(52) = −1.24, p = 0.22
Rapid automatized naming: letters (time in seconds)	28.70 (6.66)	31.81 (10.27)	t(52) = −1.32, p = 0.19
Selective attention (number of correct responses)	63.00 (9.33)	63.30 (13.53)	t(52) = −0.09, p = 0.93

#### Control group

Sixteen children (15 boys, one girl), mean age 11.5 years (range 8.8–14.0) participated in the project as a dyslexic control group. All members of the dyslexic control group were recruited in the same manner as the participants of AVG and PNAVG trainings and participated in the same broader study on dyslexia. The inclusion criteria for the control group were the same as in the case of experimental groups, i.e. (i) diagnosis of dyslexia confirmed with a standardized battery of tools performed by an experimenter involved in the project, (ii) no history of psychiatric or neurological disease, (iii) IQ ≥ 85, (iv) normal or corrected to normal vision, (v) no ADHD diagnosis. The only difference was that the members of the control group were not able to arrive at the place of training, mostly because they did not live in Warsaw where the training was conducted. Therefore, the control group did not participate in the pre-test and post-test at the place where the training was conducted, and during the training period it took part in the web-based assessment only.

However, within the broader study on dyslexia, the control group, as well as both training groups, was assessed with an intelligence test and a standardized battery for diagnosis of dyslexia. These tests showed that the control group did not differ from the training groups in terms of age, intelligence (as assessed with Wechsler Intelligence Scale – Revised^37^), reading speed and accuracy, phonological awareness and rapid automatized naming as assessed with a standardized battery for diagnosis of dyslexia^38^ (see Table 2).

**Table 2:** AVG, PNAVG and dyslexic control group characteristics. Mean (SD).

	AVG (n = 27)	PNAVG (n = 27)	CON (n = 16)	
Age (years)	11.04 (1.00)	10.96 (1.00)	11.51 (1.55)	F(2,67) = 1.27, p = 0.29
WISCR IQ	114.19 (13.10)	112.19 (11.03)	114.94 (9.84)	F(2,67) = 0.34, p = 0.71
Word reading (accuracy)^a^	2.85 (1.23)	2.78 (1.22)	2.94 (1.39)	F(2,67) = 0.08, p = 0.92
Pseudoword reading (speed)^a^	2.74 (1.16)	2.56 (1.12)	2.56 (0.73)	F(2,67) = 0.25, p = 0.78
Phonological awareness (accuracy)^a^	3.52 (1.40)	3.07 (1.77)	3.13 (1.92)	F(2,66) = 0.53, p = 0.59
Phoneme deletion (accuracy) ^a^	3.48 (1.72)	3.44 (1.76)	3.94 (1.65)	F(2,67) = 0.47, p = 0.63
RAN^b^: objects and colours (speed)^a^	3.04 (1.95)	2.96 (1.79)	3.13 (2.00)	F(2,67) = 0.04, p = 0.96
RAN^b^: digits and letters (speed)^a^	3.52 (2.05)	2.70 (1.59)	3.81 (1.72)	F(2,67) = 2.30, p = 0.11

*Notes:* ^a^Standard ten scores (sten) are reported. Population mean equals 5.5 (2.0).

^b^RAN – Rapid automatized naming.

### Apparatus, Stimuli and Procedure

#### Training procedure

The training procedure was very similar to the one used by Franceschini *et al*.^19,20^ and differed only in the equipment used for playing and exact duration of the training. Participants were tested 1 to 18 days (M = 9.2, SD = 4.4) before the start of the training and re-tested between 1 and 18 days (M = 8.0, SD = 5.1) after the end of the training. The training composed of 16 training sessions, each lasting 50 minutes (800 minutes, i.e. 13.3 hours of training in total), was performed by children in 22 to 36 days (M = 28.9, SD = 2.1). The whole programme, from the first to the last testing, took 31 to 62 days (M = 46.1, SD = 6.6).

Video games were played on 14-in laptops equipped with mouses and headphones. When playing, children were seated in a room either alone or with two to eight other participants of the training. Children from AVG and PNAVG groups were aware that the other group played other games.

AVG group played the same commercial video game from Ubisoft^TM^ as in the previous research^19,20^, called *Rayman Raving Rabbids*. Only the minigames selected as action games by Franceschini *et al*.^19^ were used in the group. As the playing sessions were supervised by experimenters, they watched the monitors of the participants and made sure that children were playing only the action minigames.

PNAVG group played six minigames based on phonological awareness and not including any components of action video games^39^ (see Supplementary materials 3). The minigames were designed exclusively for the aims of the training. The games used either pictures or words and pseudowords presented orally. In the particular minigames children were asked to: (i) pick the objects of a given phonological feature, e.g. words beginning with a given phoneme, (ii) pair the objects on the basis of a phonological rule, e.g. rhyming words, (iii) pair pseudowords on the basis of a phonological rule, e.g. words ending with the same phoneme, (iv) compose a heard pseudoword from single phonemes or syllables, (v) blend syllables of two heard pseudowords, (vi) provide a letter or syllable which differed between two auditorily presented words or pseudowords. Detailed description of the phonological games is provided as Supplementary materials 3.

#### Tasks administration and evaluation

Standard reading, phonological and attentional tasks were administrated individually during 60-minute-long sessions which incorporated behavioural (around 30 minutes) and MRI testing (not analysed in the current paper). The experimenters were unaware of participants’ allocation to AVG or PNAVG groups.

Both reading tasks, phoneme deletion and vowel replacement tasks had two variants (A and B) that differed with respect to items, but not the procedure (see Supplementary materials 1). The order of the variants was counterbalanced across participants and time points, i.e. half of the participants completed variant A before the training and variant B after it, and the second half started from variant B and completed variant A after the training.

#### Reading tasks

Children were presented with lists of words or pseudowords of increasing length. There were two lists of words (containing 75 items each), and two lists of pseudowords (containing 69 items each). Participants were asked to read aloud as many words as possible in 30 seconds, then the procedure was repeated for the second list. Variants A and B differed in the order of the two lists. The total number of correctly read items in a minute (sum of the two lists) and the number of read syllables were calculated for each child for words and pseudowords separately.

#### Phonological tasks

Phoneme deletion: Children were asked to repeat a real word with one phoneme deleted (e.g. /kɔva/ without /r/ is /kɔva/). The list contained 16 items, and variants A and B differed in terms of exact items. Both accuracy and time necessary to finish the task (in seconds) were considered in the analyses.

Vowel replacement: Children were asked to repeat a real word, replacing all occurrences of the /a/ vowel with either /ɛ/ or /u/ (depending on the variant of the task, A or B). The list incorporated 8 one-syllable words (e.g. /park/ changed to /purk/ or /pɛrk/) and 8 two-syllable words (e.g. /brama/ changed to /brumu/ or /brɛmɛ/). Both accuracy of 24 replacements and time necessary to complete the task were calculated for the analyses.

Pseudoword repetition: A standardized pseudoword repetition task was applied^40^. The list contained 27 pseudowords and the number of correctly repeated pseudowords was calculated for each child.

#### Attentional tasks

Selective attention: A subtest of IDS Intelligence Scale^28^ was applied to test selective attention. The task comprised 225 pictures of ducks, arranged in 9 lines of 25 pictures. Children were required to cross out all the ducks which were looking to the right and which had exactly two body parts coloured in orange. There were about 10 target ducks in each line and children could not spend more than 15 seconds per line. The difference between the number of correctly and incorrectly crossed out ducks was treated as a measure of selective attention.

*Rapid automatized naming*: A standardized battery of four rapid naming tasks was applied^41^. The battery consisted of boards of objects, colours, digits and letters. There were eight lines of six items printed on each board. The time of naming all 48 items was measured separately for each board.

#### Web-based reading tasks: word recognition, sentence reading and decoding

In addition to the aforementioned reading tests, three web-based tasks were designed for the purpose of assessment of progress in reading-related functions across AVG, PNAVG and the dyslexic control groups. The three web-based tests were characterized by high reliability in terms of Cronbach’s alpha (Word recognition task: α = 0.88; Sentence reading task: α = 0.94; Decoding task: α = 0.71).

Word recognition task: In the first reading task (word vs nonword recognition), children were asked to click on the real word in each line (e.g. “własny” is a real Polish word, whereas “właspy” and “młasny” are not).

Sentence reading task: In the second (reading comprehension) task children read short and simple sentences and were asked to assess whether they were true or false.

Decoding task: The third task measured phonological decoding: children read three pseudowords and were asked to click on the one that is pronounced differently from the two others (e.g. “fidzka” and “ficka” are pronounced in the same way (i.e. are pseudohomophones), whereas “fizka” sounds differently).

All the tasks had a strict time limit (differing across the three tasks) not allowing children to complete all the items. Each task had four iterations, i.e. incorporated four screens appearing one after another. The total number of correct responses provided before the time limit, summed for the four iterations, was used as a measure of performance. Detailed description of the online reading tasks is provided as Supplementary materials 2.

Participants completed the tasks online working from home. Children who participated in the training completed the tasks approximately a month before the training (18–38 days, M = 30.0, SD = 6.1), then in the week preceding the training (0–7 days, M = 1.5, SD = 1.5), then again closely after the training (0–18 days, M = 4.8, SD = 4.5) and, finally, around a month after the training (20–60 days, M = 40.9, SD = 6.9). The control group completed the tasks on four occasions approximately one month (16–60 days, M = 38.3, SD = 10.8) apart. In order to compare directly the effect of training across the three groups, only data from the second and third testing (i.e. directly before and directly after the training) is analysed in the main text and data from all four testings in analysed in Supplementary material 4.

### Data availability

The datasets generated and analyzed during the current study are available from the corresponding author on request.

## Electronic supplementary material

Supplementary materials
