# Supplementary material for: Neither action nor phonological video games make dyslexic children read better
Source: Sci Rep. 2018 Jan 11;8:549. doi: 10.1038/s41598-017-18878-7 (PMC5765029; doi:10.1038/s41598-017-18878-7)
Supplement: Supplementary file 1 — Supplementary materials [file 41598_2017_18878_MOESM1_ESM.doc]

# Neither action nor phonological video games make dyslexic children read better: Supplementary materials

Magdalena Łuniewska^1^, Katarzyna Chyl^1^, Agnieszka Dębska^1^, Agnieszka Kacprzak^1^, Joanna Plewko^1^, Marcin Szczerbiński^2^, Jakub Szewczyk^3^, Anna Grabowska^1,4^, Katarzyna Jednoróg^1, *^

^1^ Laboratory of Psychophysiology, Department of Neurophysiology, Nencki Institute of Experimental Biology, Polish Academy of Sciences, Warsaw, Poland

^2^ School of Applied Psychology, University College Cork, Cork, Ireland

^3^ Institute of Psychology, Psychology of Language and Bilingualism Lab, Jagiellonian University, Kraków, Poland

^4^ Faculty of Psychology, University of Social Sciences and Humanities, Warsaw, Poland

***Corresponding author:**

Katarzyna Jednoróg

Laboratory of Psychophysiology, Department of Neurophysiology,

Nencki Institute of Experimental Biology, Pasteur 3, 02-093 Warsaw, Poland

Email: [k.jednorog@nencki.gov.pl](mailto:k.jednorog@nencki.gov.pl)

Phone: +48 22 5892 392; Fax: +48 22 5892 490

# Supplementary materials 1: the tasks used for assessment of reading and phonological skills

## Reading tasks

### Word and pseudoword reading

The task was a part of a standardized test battery for early detection of reading problems (Szczerbiński & Pelc-Pękala, 2013). Children were presented with lists of words (75 items) or pseudowords (69 items) of increasing length and orthographic complexity and asked to read aloud as many words as possible in 30 seconds. The instruction was as follows: “Read as many words from this list as possible. Read from top to bottom and do not skip any word. Read as quickly and as correctly as possible. You will have only half a minute for that.”. After 30 seconds the experimenter said: “Stop”, and repeated the procedure for the second list. Variants A and B differed in the order of the two lists. The order was List I – List II in the variant A, and List II – List I in the variant B. The total number of correctly read items in a minute (sum of the two lists) and the number of read syllables were calculated for each child for words and pseudowords separately.

## Phonological awareness tasks

### Phoneme deletion

This task was adapted from a standardised test battery for the assessment of dyslexia (Bogdanowicz et al., 2009). Children were asked to repeat a real word with one phoneme deleted. The list contained 16 items, and variants A and B used different sets of words. The instruction was as follows: “You will be a phoneme[[1]](#footnote-2)-eater now. I will tell you a word and will ask you to eat a given phoneme, and you will tell me what is left from the word. For example, I will say /d[ɔ](https://en.wikipedia.org/wiki/Open-mid_back_rounded_vowel)m[ɛ](https://en.wikipedia.org/wiki/Open-mid_front_unrounded_vowel)k/[[2]](#footnote-3) eat /*d*/ then you will say /[ɔ](https://en.wikipedia.org/wiki/Open-mid_back_rounded_vowel)m[ɛ](https://en.wikipedia.org/wiki/Open-mid_front_unrounded_vowel)k/ because /d[ɔ](https://en.wikipedia.org/wiki/Open-mid_back_rounded_vowel)m[ɛ](https://en.wikipedia.org/wiki/Open-mid_front_unrounded_vowel)k/without /*d/* gives /[ɔ](https://en.wikipedia.org/wiki/Open-mid_back_rounded_vowel)m[ɛ](https://en.wikipedia.org/wiki/Open-mid_front_unrounded_vowel)k/If I say /d[ɔ](https://en.wikipedia.org/wiki/Open-mid_back_rounded_vowel)m[ɛ](https://en.wikipedia.org/wiki/Open-mid_front_unrounded_vowel)k/ without /*k*/, you will say /d[ɔ](https://en.wikipedia.org/wiki/Open-mid_back_rounded_vowel)m[ɛ](https://en.wikipedia.org/wiki/Open-mid_front_unrounded_vowel)/ because /d[ɔ](https://en.wikipedia.org/wiki/Open-mid_back_rounded_vowel)m[ɛ](https://en.wikipedia.org/wiki/Open-mid_front_unrounded_vowel)k/without /*k/* gives /d[ɔ](https://en.wikipedia.org/wiki/Open-mid_back_rounded_vowel)m[ɛ](https://en.wikipedia.org/wiki/Open-mid_front_unrounded_vowel)/Let’s try it!” This instruction was followed by three training trials with immediate feedback, whereas there was no feedback in the test trials. Both accuracy and time necessary to finish the whole task (in seconds) were included in the analyses. All test items were real words, while a product of correct phoneme deletion was always a pseudoword. The position of the target to-be-deleted consonant varied across items (word initial, word final, middle of word; single or embedded within a consonant cluster).

### Vowel replacement

In this task children were asked to repeat real words containing vowel /a/, replacing that vowel with /u/ (variant A) or /ɛ/ (variant B) (Szczerbiński, 2001). The list incorporated 8 one-syllable words and 8 two-syllable words, all containing vowels /a/. The instruction for variant A was as follows: “Let’s try a secret language now. It will be called ATU because instead of the sound /a/ we will say /u/. Every time when you hear /a/ you will change it to /u/. Instead of /las/ you will say /lus/, instead of /ja/ you will say /ju/, instead of /mam/ – /mum/. What will you say instead of /xak/?... And instead of /dax/?... And /pla[t͡s](https://en.wikipedia.org/wiki/Voiceless_dental_sibilant_affricate)/”… Corrective feedback was given only on training trials. After the 8 one-syllable words, the instruction was repeated: “Now try to do the same thing with the words in which there are two /a/ sounds. You have to replace both of them with /u/. Instead of /mama/ you will say /mumu/, instead of /tata/ – /tutu/, instead of /vata/…, and instead of /kara/…?”. Both accuracy of 24 replacements and time necessary to complete the whole task (both parts) were calculated for the analyses.

# Supplementary materials 2: the web-based reading tasks

## Overview

Web-based reading tasks were designed in order to enable the comparisons with the control group, as the control participants were children who were not able to travel to the place of training and testing.

Children in both training groups completed all tests four times at monthly intervals: one month before the training, just before the beginning of the training, just after the training was finished, and one month after the end of the training. The dyslexic control group completed all tests four times one month apart each other.

In this paper, only data from two middle measurements were analysed: T1 – directly before the training and T2 – directly after the training, as these corresponded to time points of other tests. The web-based tasks were completed online, during leisure time at home. It took approximately 15 minutes to complete one testing session.

All three reading tasks came from an experimental paper-and-pencil reading test battery (Szczerbinski, 2011), with adaptations necessary to make it suitable to online presentation.

## Instruction

### General instructions for parents

Before each session, parents received a remainder with the detailed instructions on how to perform the testing session. The reminder was worded as follows:

“We would like to remind you that it is crucial that every child completes the testing session in similar conditions. Therefore, we ask you to strictly follow the instructions below:

1. To carry out the testing session, you need a computer with an internet connection and a computer mouse. The testing should not be done on a tablet or a smartphone. Using a computer mouse enables children to give the answers quicker and makes it possible to test their reading abilities in a more reliable way.
2. We encourage you to carry out the testing when the child is relaxed, focused and willing to work.
3. The surroundings should be quiet and there should be only the child with a parent (or a caregiver) in the room.
4. All tasks have a time limit and the child will get only one attempt to complete the test.
5. Children should do the tasks without any help. They should read the short instructions before each task themselves. However, a parent (or a caregiver) may stay nearby to provide some help in case of any technical difficulties (e.g. disconnection to the internet or an accidental shutting down of the web browser) and to check whether all the tasks are completed.
6. The parent should not help the child in any other way (especially by prompting the correct answers, or indicating the incorrect answers). Only a session completed by the child himself/herself provides reliable and useful results.
7. When all tasks are finished, the completion message will be displayed. We encourage to complete all tasks at one sitting but in case of technical problems it will be possible to return to the test and finish it.

Please contact us if you have any questions or doubts.”

### General instruction for children

After opening the appropriate website (linked to the child’s individual account) the following instruction for the child was displayed: “In a moment we will ask you to complete five short tasks. Each of them will be displayed in several parts. Try to work as fast as you can. When you click the ‘Start’ button, the first task will begin and you will work on your own, without any help from your parent.”

## Tasks description

The testing session consisted of five tasks. Three tasks which were included in the current analysis are described in details below. The other two were a training task (in which children’s aim was to pick a smiled face in each line) and an orthographic sensitivity task which was not included in analysis as not assessing reading-related skills directly.

Each test was preceded with three to five training trials with immediate feedback, whereas there was no feedback in the test trials. Each task described below consisted of four trials with 10 items in each trial. Every trial was followed by the massage: “Good job! Now try to work even faster”, and a “Start” button which allowed to begin the next trial.

### Word recognition

In each trial, three words were displayed simultaneously on screen. One was a real word of high frequency in Polish (e.g. “własny”) and two others were pseudowords created by substituting a single letter of the target (e.g. “właspy” and “młasny”). Child’s task was to choose the real word. The instruction was as follows: “You will see some real and some fake words. In each line, there will be only one real word. Click on the real words. Work as fast as you can!”. The time limit for each trial was set to 20 seconds. The score was the total number of correctly chosen words across all four trials.

### Sentence comprehension

The items were short sentences that were either clearly true or clearly false, e.g.: “Słońce świeci w dzień” (The sun shines during the day) or “Bociany żywią się cukierkami” (Storks feed on candies). The child’s task was to make the correct decision. The exact instruction was: “You will read some sentences. Choose whether the sentence is true or false.” The time limit for each trial was set to 30 seconds. The score was the total number of correct answers across all four trials.

### Decoding

In each trial, three pseudowords were displayed on screen. Two of them had identical pronunciation in Polish (i.e. they were pseudohomophones, e.g. “ficka” and “fidzka” both pronounced as /fi[t͡s](https://en.wikipedia.org/wiki/Voiceless_dental_sibilant_affricate)ka/), the third (e.g. “fiska” pronounced as /fiska/) differed in pronunciation by one phoneme only (i.e. it was a minimal pair of the other two). The child’s task was to choose the pseudoword that was pronounced differently. The instructions were as follows: “Look at these words: TUK, TÓK, TUD. Two of them are pronounced the same way and one of them is pronounced differently. The word TUD is pronounced differently. The words TUK and TÓK are pronounced the same way, although they are written differently. In each line find the word that is pronounced differently than the two others.” The time limit for each trial was set to 30 seconds. The score was the total number of correctly chosen pseudowords across all trials.

# Supplementary materials 3: the phonological awareness non-action video games (PNAVG)

## Overview

Phonological awareness training was designed as a non-action computer game about dragons. We aimed to create a game appealing for children to keep their motivation high, and thus colorful animations and solutions known from commercial computer games for children were employed (e.g., for correctly solved items gold coins were given). The training was planned for 16 hours, and divided into 16 one-hour training sessions. However, actual games were played for 50 minutes, while in remaining time children could spend the gold coins earned for the previous in-game achievements.

## Game types

Phonological awareness training consisted of six basic game types. On each training session, a child could play two to four minigames from a given game type, each lasting 2 to 4 minutes (8 minutes per minigame type in total). The number and the type of minigames that children were allowed to play were pre-defined, but children could freely choose their order. Before the first session, detailed instructions for each game type were given. Afterwards, throughout the training and before each minigame, short instructions were provided.

The training was purely auditory. The items consisted of words (occasionally accompanied by pictures depicting their meaning) and pseudowords. Overview of the types of games is given below.

### Chinese Dragon

Item types: pictures coupled with pictures names.

Instruction example: “Eat items starting with /m/”.

Game was designed as similar to the popular “Snake” game. The task of the player controlling Chinese Dragon was to eat targets by running into them, and to avoid distractors. If the chosen item matched the criteria, the player gained one gold coin and the Chinese Dragon grew.

### Dragon Eggs

Item types: pictures coupled with pictures names

Instruction example: “Choose items pairs with equal number of syllables”

Game was inspired by the popular card game known as Concentration, Memory or Pairs. A player attempts to find the matching pairs by clicking on the dragon eggs and revealing the objects hidden underneath. If the paired objects match according to the instruction, eggs disappear and a player gains three gold coins.

### Two-Headed Dragon

Item types: pictures coupled with pictures names; words; pseudowords

Instruction example: “Type a letter that is in the first word, and is missing in the second word”

A player attempts to solve the riddles asked by the Two-Headed Dragon. Each dragon’s head produces one word or pseudoword, and the player’s task is to type a letter or few letters that differentiate them according to the instruction.

It is the only game from the training in which a player is asked to actively produce the answer by typing it on a computer keyboard. Items selected for this game were orthographically transparent to avoid confusion caused by differences between phonology and orthography.

### Dragon Babies

Item types: pseudowords

Item example: parents: /k[ɔ](https://en.wikipedia.org/wiki/Open-mid_back_rounded_vowel)r[ɛ](https://en.wikipedia.org/wiki/Open-mid_front_unrounded_vowel)/, /luta/; targets: /k[ɔ](https://en.wikipedia.org/wiki/Open-mid_back_rounded_vowel)ta/, / r[ɛ](https://en.wikipedia.org/wiki/Open-mid_front_unrounded_vowel)lu/; distractors: /k[ɔ](https://en.wikipedia.org/wiki/Open-mid_back_rounded_vowel)tun/, /piki/

Instruction example: “Match the dragon babies with their parents”

Babies’ names are a combination of their parents’ names. A player attempts to match the dragon babies and their parents by dragging & dropping eggs to their parents’ nest. For each correctly identified egg, player gains one gold coin.

### Dragon Flirt

Item types: pseudowords

Instruction example: “Match the dragons whose names rhyme”

A player’s task is matchmaking the dragons according to the rule presented in the instruction. By dragging and dropping selected dragon to its mate, a player matches the couples. If the instruction criteria were fulfilled and the dragon couple match, player gains one gold coin, and the couple disappears.

### Magician

Item types: pseudowords

Instruction example: “Choose the ingredients to make the magic potion”

A magic potion (a pseudoword) consists of several ingredients (phonemes or syllables). A players’ task is to reconstruct the formula and choose all the ingredients needed for the potion. If all the ingredients were chosen correctly and placed in order in the cauldron, a player gains two gold coins.

## Items

Pseudowords were generated from n-grams selected from words included in the National Corpus of Polish. We first generated a pool of nonwords that were maximally varied with respect to length and n-gram frequency, and then selected some of them such that they matched the requirements of specific levels of difficulty (see below). N-gram frequency is an index of phonotactic probability that is calculated as the mean (log) frequency of all ngrams that occur in a given (pseudo-)word. Our prior research has shown superiority of this index over any other indices of phonotactic probability in predicting the difficulty of pseudoword phonological processing (Szewczyk, Marecka, Chiat, & Wodniecka, in review).

## Difficulty levels

The difficulty level for each game was estimated on the basis of informal tests of the games. We manipulated item types (pictures with names, words, pseudowords), complexity (word frequency for pictures with names and words, n-gram frequency for pseudowords), and length (number of syllables and phonemes), depending on the task in the particular minigame. The number of items available on the given level could vary (e.g. in Dragon Eggs, Dragon Babies, Dragon Flirt), as well as the target–to-distractor ratio (e.g. in Chinese Dragon, Dragon Eggs, Dragon Babies or Magician). An important factor determining the difficulty level in each minigame was the exact type of task given to children. For example, we assumed that finding pseudowords that rhyme in Polish is easier than finding pseudowords with certain number of syllables in it or words with consonants in the second to last position. Additionally, we controlled the time constrain for each task in each game. The more advanced the player, the stricter was the time limit.

## Adaptivity

Each of the six game types used in the training contained 32 to 64 minigames of growing difficulty level. Additionally, each minigame had three parallel versions, with the same parameters (e.g. time limits) and instructions, but different or partially different items. The player could level up if 75% of responses were correct. If less than 75% of items were solved, the player had to repeat a given level, but no more than two extra times; after three failures, the game leveled up automatically. However, players were never informed if the game was completed successfully or not, and what is their progress. The only explicit measure of progress available was the number of gold coins collected in a given session.

# References

Bogdanowicz, M., Jaworowska, A., Krasowicz-Kupis, G., Matczak, A., Pelc-Pękala, O., Pietras, I., Stańczak, J., & Szczerbinski, M. (2009). *Diagnoza dysleksji u uczniów kl. III szkoły podstawowej. Przewodnik diagnostyczny* [Assessment of dyslexia in the 3^rd^ grade of primary school: A diagnostic guide]. Warszawa: Pracownia Testów Psychologicznych.

Szczerbinski, M. (2011). *Grupowy Test Czytania-PL* [Group Reading Test-Polish]. Unpublished manuscript, University College Cork.

Szczerbinski, M. (2001). *Learning to read and spell single words: a case study of a Slavic language*. Unpublished PhD dissertation. University College London.

Szczerbinski, M., Pelc-Pekala,O. (2013) *Zestaw metod do diagnozy trudności w czytaniu – Test Dekodowania* [The Decoding Test – a set of tools for diagnosing reading difficulties]. Gdansk: Pracownia Testów Psychologicznych i Pedagogicznych.

Szewczyk J., Marecka M., Chiat S., Wodniecka Z. (under review) Nonword repetition depends on the frequency of sublexical representations at different grain sizes: evidence from a multi-factorial analysis

# Supplementary materials 4: Reading improvements as compared to the dyslexic control group across four time-points

The aim of this additional analysis is to investigate the effects of Time (time: a month before training, directly before training, directly after training, a month after training) and Group (AVG vs. PNAVG vs. CON) on the performance in three web-based tests: (1) word recognition, (2) speed of sentence reading, and (3) speed of decoding. We performed three separate 4 x 3 ANOVAs with a within-subject factor of Time (a month before training, directly before training, directly after training vs. a month after training) and a between-subject factor of Group (AVG vs. PNAVG vs. CON), and the number of correct responses given in a limited time in each task as the dependent variable.

Main effects of time were significant for speed of sentence reading and for speed of decoding (see Supplementary Supplementary Table 1): in both tasks the scores increased with time (see Supplementary Supplementary Figure 1). There was no significant effect of group, i.e. AVG, PNAVG and the control group did not differ systematically in terms of performance in the tasks. There was no significant effect of Time * Group interaction, i.e. the progress in solving the online tasks did not differ between the three groups.

Supplementary Table 1. Main effects of Time (four time-points), Group (AVG vs. PNAVG vs. control) and Time * Group interaction in the three web-based tests.

|  | Time | Group | Time * Group |
| --- | --- | --- | --- |
| Word recognition | F(3,54) = 2.38, p = .08,  η^2^ = .12 | F(2,56) = 0.58, p = .56 | F(6,110) = 2.12, p = .06, η^2^ = .10 |
| Speed of sentence reading | F(3,54) = 6.28, p < .001,  η^2^ = .26 | F(2,56) = 0.72, p = .49 | F(6,110) = 0.68, p = .66 |
| Speed  of decoding | F(3,53) = 3.38, p = .03,  η^2^ = .16 | F(2,55) = 0.42, p = .66 | F(6,108) = 0.68, p = .66 |


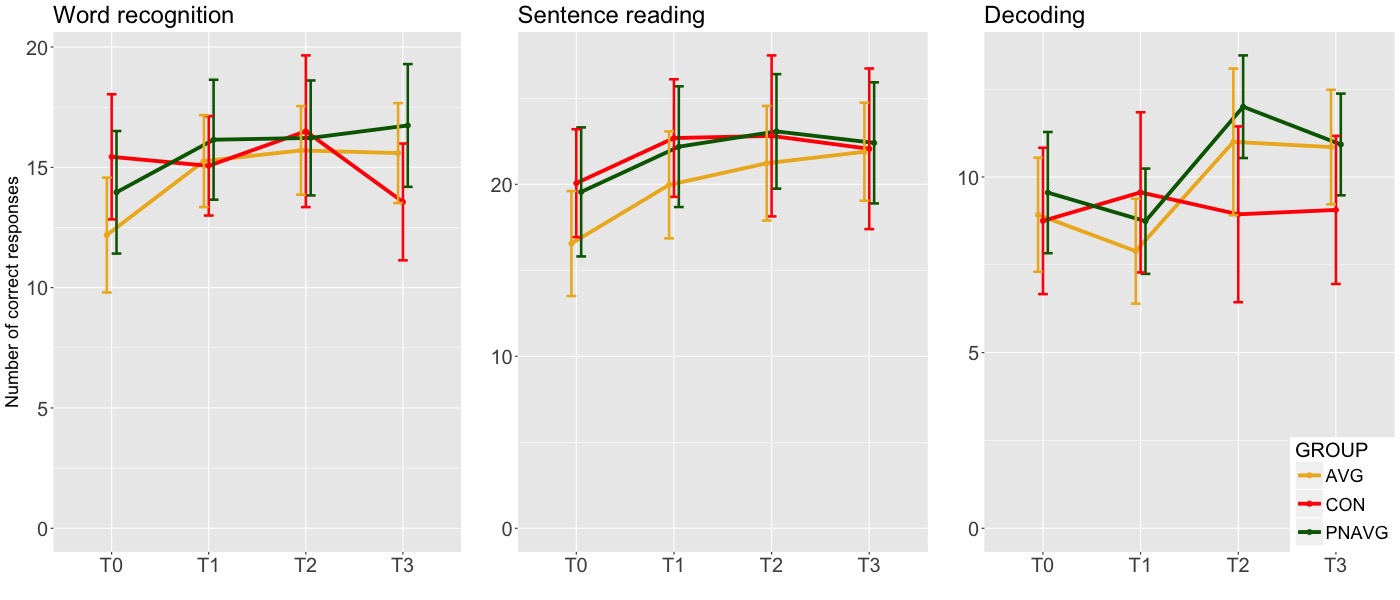


Supplementary Figure 1. Progress in online reading tasks between T0 (a month before the training), T1 (directly before the training), T2 (directly after the training) and T3 (a month after the training) in AVG (yellow line), PNAVG (green line) and control group (red line). Error bars correspond to 95% confidence intervals. In speed of sentence reading and speed of decoding, there were significant effects of Time. In all tasks, there was neither effect of Group nor Group * Time interaction.

1. The children were familiar with the notion of the ‘phoneme’. [↑](#footnote-ref-2)
2. We used simplified IPA (International Phonetic Alphabet) transcript to transcribe words presented to children orally. [↑](#footnote-ref-3)
